# Solid Phase Synthesis of Mitochondrial Triphenylphosphonium-Vitamin E Metabolite Using a Lysine Linker for Reversal of Oxidative Stress

**DOI:** 10.1371/journal.pone.0053272

**Published:** 2013-01-14

**Authors:** Mohanad Mossalam, Jamie Soto, Carol S. Lim, E. Dale Abel

**Affiliations:** 1 Department of Pharmaceutics and Pharmaceutical Chemistry, University of Utah, Salt Lake City, Utah, United States of America; 2 Program in Molecular Medicine and Division of Endocrinology, Metabolism and Diabetes, University of Utah School of Medicine, Salt Lake City, Utah, United States of America; University of Tor Vergata, Italy

## Abstract

Mitochondrial targeting of antioxidants has been an area of interest due to the mitochondria's role in producing and metabolizing reactive oxygen species. Antioxidants, especially vitamin E (α-tocopherol), have been conjugated to lipophilic cations to increase their mitochondrial targeting. Synthetic vitamin E analogues have also been produced as an alternative to α-tocopherol. In this paper, we investigated the mitochondrial targeting of a vitamin E metabolite, 2,5,7,8-tetramethyl-2-(2′-carboxyethyl)-6-hydroxychroman (α-CEHC), which is similar in structure to vitamin E analogues. We report a fast and efficient method to conjugate the water-soluble metabolite, α-CEHC, to triphenylphosphonium cation via a lysine linker using solid phase synthesis. The efficacy of the final product (MitoCEHC) to lower oxidative stress was tested in bovine aortic endothelial cells. In addition the ability of MitoCEHC to target the mitochondria was examined in type 2 diabetes db/db mice. The results showed mitochondrial accumulation *in vivo* and oxidative stress decrease *in vitro*.

## Introduction

Endothelial dysfunction in diabetic patients is mainly caused by hyperglycemia, which increases reactive oxygen species (ROS) production in mitochondria [1], [2], [3]. Myocardial mitochondria are involved in the generation of energy, the regulation of apoptosis, and the production and detoxification of ROS [4], [5], [6]. Disrupting the mitochondrial Ca^2+^, ATP, or ROS metabolism plays a role in different diseases namely diabetes, obesity, heart failure, stroke, aging, cancer, and neurodegenerative diseases [7], [8], [9]. To diminish ROS production, the cell has its own antioxidant defenses such as glutathione, catalase, and superoxide dismutase to prevent oxidative stress [10]. Maintaining ROS/antioxidant ratio is imperative for cell signaling [11]. Since the mitochondria produces and metabolizes ROS, targeting antioxidants to the mitochondria has been a focus of interest. This is achieved by conjugating an antioxidant to a lipophilic cation. The positive charge enables mitochondrial accumulation 100–1000 times higher due to the high inner mitochondrial negative membrane potential, 160–185 mV [12], [13], [14].

Conjugating an antioxidant such as vitamin E to a lipophilic cation can be a promising approach for reversing oxidative damage [15], [16]. Vitamin E is a fat-soluble antioxidant with eight naturally occurring vitamer forms. The most common forms are γ- and α-tocopherol in human plasma and tissues [17]. Vitamin E has a positive effect on insulin sensitivity and the prevention of type-2 diabetes [18], [19], [20] due to its antioxidant capacity [21], [22]. Alpha-tocopherol is a chain-breaking antioxidant which interrupts the formation of lipid-derived oxygen- and carbon-centered free radicals by preventing the propagation step [11]. These radicals are formed via a chain reaction: initiation, propagation, and termination [23]. High intake of vitamin E has been associated with lower risk for coronary heart disease [24], [25], [26]. Despite reported success, mixed results have been reported regarding the recommendations of vitamin E supplement as a prevention or treatment of cardiovascular disease [27], [28], which could be due to poor uptake and lack of mitochondrial accumulation [29], [30]. Targeting vitamin E conjugated to triphenylphosphonium (TPP^+^) to the mitochondria has been reported to decrease ROS better than vitamin E itself [31], [32].

Synthetic vitamin E analogues have been produced as an alternative to tocopherol for cardiovascular therapy [33], [34], [35]. A major water-soluble metabolite of α-tocopherol with a structure similar to these analogues is 2,5,7,8-tetramethyl-2-(2′-carboxyethyl)-6-hydroxychroman (α-CEHC), which is normally detected in human blood and urine after vitamin E supplementation [36], [37]. It has been proposed that α-CEHC is exclusively formed in hepatic mitochondria via ω-hydroxylation of α-tocopherol to 13′-OH-α-tocopherol in hepatic microsomes followed by five rounds of β-oxidation in both the peroxisomes and mitochondria [38]. Importantly, α-CEHC can also act as an antioxidant similar to trolox, a water-soluble derivative of vitamin E [39]. Due to water-solubility and antioxidant activity of α-CEHC, we choose to conjugate TPP^+^ to this particular analogue of vitamin E.

This study reports the synthesis of a novel α-CEHC-TPP^+^ conjugate (MitoCEHC) with an improved conjugation method using a lysine linker and solid phase synthesis. We also tested the efficacy of the new conjugated MitoCEHC in decreasing ROS in bovine aortic endothelial cells (BAECs) and in targeting the mitochondria in type 2 diabetic db/db mice [40].

## Materials and Methods

### Ethics statement

All animal experiments were conducted in strict accordance with the recommendations in the Guide for the Care and Use of Laboratory Animals of the National Institutes of Health. The protocol was approved by the Institutional Animal Care and Use Committee (IACUC) at the University of Utah (permit number 09-08011).

### Synthesis of (4-(((2R)-1-amino-6-(3-(hydroxyl-2,5,7,8-tetramethylchroman-2-yl)propanamido)-1-oxohexan-2-yl)amino)-4-oxobutyl)triphenylphosphonium (α-CEHC-lysine-TPP^+^, MitoCEHC or 8)

**Figure 1:**
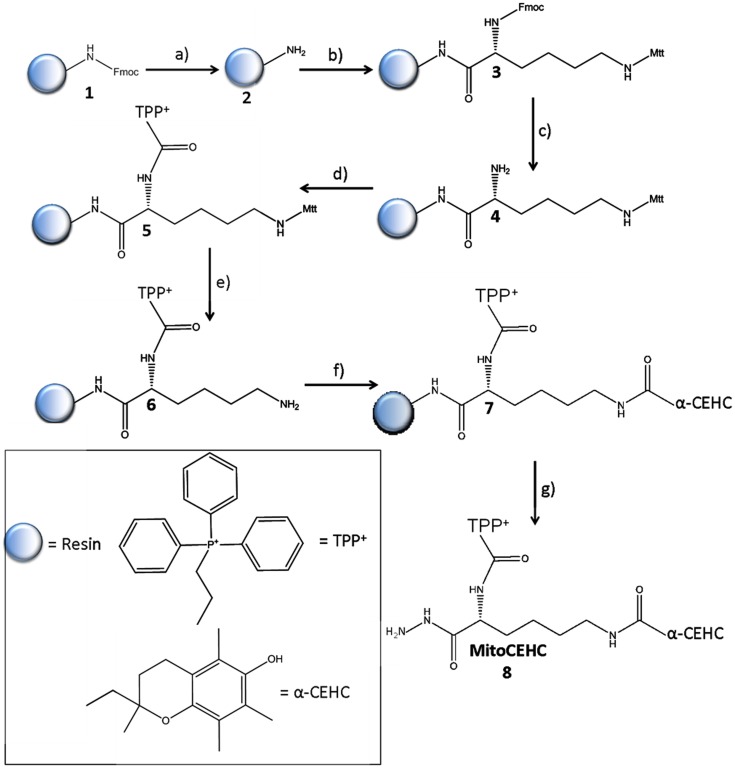
Solid phase synthesis of MitoCEHC (8). *Reagents and conditions:* a) 20% piperidine, DMF. b) Fmoc-Lys[Mtt]-OH, HBTU, HOBt, DIPEA, DMF. c) 20% piperidine, DMF. d) (3-carboxyproppyl)TPP^+^, HBTU, HOBt, DIPEA, DMF. e) 94% DCM, 5% Tis, 1% TFA. f) α-CEHC, HBTU, HOBt, DIPEA, DMF. g) 95% TFA, 2.5% water, 2.5% Tis.

MitoCEHC was prepared as shown in Figure 1. A lysine linker with two protecting groups (Fmoc and Mtt) was used, which enabled the conjugation of TPP^+^ and α-CEHC. The Rink Amide resin (EMD Chemicals, Germany) containing Fmoc (**1**) (0.02 mmol) was dissolved in dimethylformamide (DMF), loaded into a fritted column (Grace Davison Discovery Sciences, Deerfield, IL) and washed twice in DMF [41]. The Fmoc was deprotected with 20% piperidine (Sigma-Aldrich, St. Louis, MO) in DMF for 15 minutes at room temperature. After deprotection was complete, the reaction column was drained and washed with DMF. In a separate tube, 3 equivalents of Fmoc-Lys[Mtt]-OH (Anaspec, Fremont, CA) was added to 5 equivalents of HBTU (EMD Chemicals), 5 equivalents of HOBt (EMD Chemicals), and 5 equivalents of *N*,*N*-Diisopropylethylamine (DIPEA, Sigma-Aldrich) in DMF. This coupling solution was then added to the resin (**2**) to couple the lysine. The mixture was agitated for 5 hours at room temperature. The Fmoc on the lysine (**3**) was deprotected with 20% piperidine in DMF as mentioned above. Subsequently, 5 equivalents of (3-carboxypropyl)TPP^+^ (Sigma-Aldrich) were coupled to the lysine (**4**) via HBTU (5 equivalents), HOBt (5 equivalents), and DIPEA (5 equivalents) in DMF for 5 hours at room temperature as described above. The Mtt group on the lysine (**5**) was then de-protected by incubation with 94% dichloromethane (DCM, Sigma-Aldrich), 5% Triisopropylsilane (Tis, Sigma-Aldrich), and 1% trifluoroacetic acid (TFA, Sigma-Aldrich) for 15 minutes. Next, 3 equivalents of α-CEHC (Cayman Chemical, Ann Arbor, MI) was coupled to the lysine (**6**) via HBTU (5 equivalents), HOBt (5 equivalents), and DIPEA (5 equivalents) in DMF for 5 hours at room temperature as described above. Finally, the resin (**7**) was treated with 95% TFA, 2.5% water, and 2.5% Tis and agitated for two hours. The treatment allowed the cleavage of the final product **(8)**, which was named MitoCEHC, from the resin. The filtrate was then collected and dried for 2 hours in vacuo. The final compound (0.013 g, 87% yield) was analyzed with LC/MS and MALDI-TOF mass spectrometry. A sample of the filtrate was air-dried and then re-dissolved in methanol. Full scan spectra were recorded by scanning a *m/z* range of 500–2000.

### Cell Culture and ROS Measurement

Bovine Aortic Endothelial Cells (BAECs, Cambrex BioScience, Walkersville, MD) were grown as monolayers in DMEM (Invitrogen, Carlsbad, CA) supplemented with 10% fetal bovine serum (Invitrogen), 1% penicillin-streptomycin (Invitrogen) [6]. The cells were incubated in a 5% CO_2_ incubator at 37°C. A density of 2×10^5^ cells were seeded in 6-well plates and treated with 5 mM or 25 mM glucose [6]. The cells treated with 25 mM glucose were then incubated with 2 µM α-CEHC or 2 µM of our MitoCEHC product (**8**) for 36 hours. As before [6] the ROS production was measured after treating the cells with 10 µM 5-(and-6)-chlorodihydrofluorescein diacetate, acetyl ester (CM-H_2_DCFDA, Invitrogen), an oxidative stress indicator, for 30 minutes. CM-H_2_DCFDA is not fluorescent until the cleavage of the acetate groups via intracellular esterases occurs, followed by oxidation within the cell [42]. The oxidation of CM-H_2_DCFDA was detected by analyzing the increase in fluorescence using flow cytometry at the University of Utah Core Facility [43]. Excitation was set at 490 nm and detected at 520 nm. The experiment was repeated in triplicate (n = 3). The data was presented as the mean ± standard error. Statistical analysis was performed by using one-way ANOVA with Tukey's post test. A value p<0.05 was considered significant.

### Animal Studies and Mitochondria Isolation

Highly insulin resistant db/db mice (n = 4) were provided with 200 µM of the MitoCEHC (**8**) in their drinking water for two weeks [44], [45], [46]. Since mitochondria-targeted compounds (TPP^+^ conjugated to several antioxidants) have similar mitochondrial targeting and organ distribution when up to 500 µM is supplemented in drinking water without any gross signs of toxicity [45], 200 µM of MitoCEHC is efficient to detect mitochondrial targeting. Another population (n = 4) was given plain water (no MitoCEHC) as a negative control. After two weeks, the mice were sacrificed and their hearts were minced in STE1 buffer (250 mmol/l sucrose, 5 mmol/l Tris/HCL, 2 mmol/l EGTA, pH 7.4) and then incubated in STE2 buffer (STE1 containing [wt/vol] 0.5% BSA, 5 mmol/l MgCl2, 1 mmol/l ATP, and 2.5 units/ml protease type VIII from Bacillus licheniformis) for 4 minutes on ice to digest [44]. The mixture was then diluted in STE1 buffer and homogenized using a Teflon pestle in a Potter-Elvejhem glass homogenizer. The homogenate was centrifuged at 8000 g for 10 minutes at 4°C. Subsequently, the pellet was resuspended in STE1 buffer and centrifuged at 700 g for 10 minutes at 4°C. The pellet was discarded and the supernatant was centrifuged at 8000 g for 10 minutes at 4°C. The final mitochondrial pellet was diluted in STE1 buffer to a final concentration of 0.1 mg/ml.

### LC/MS for *in vivo* samples

The mitochondrial fraction was sonicated for 5 seconds with maximum speed in an ice bath then stirred for 30 seconds. The sonication and stirring were repeated six times. The concentrations of MitoCEHC (**8**) in the collected samples were simultaneously measured against a six-point concentration standard curve (0, 0.5, 1, 2, 4, and 8 µg/ml) using LC/MS [47]. Samples (mitochondrial fraction and plasma) and standard controls were then analyzed on the LC/MS, University of Utah Department of Chemistry. The analysis was performed by MassLynx Mass Spectrometry software (Waters Corp, Milford, MA).

## Results and Discussion

Even though mitochondria are the primary source of cellular energy, they are also the major source of ROS [48]. Therefore mitochondrial dysfunction has been under investigation more than any other organelle due to their vulnerability to oxidative damage and their contribution to apoptosis [49]. As a result of limited therapeutic accumulation within mitochondria [29], [30], [50], targeting the mitochondria with antioxidants or therapeutics has been a major interest especially for cardiovascular disease and cancer [14], [51]. Small molecules can permeate through the mitochondrial outer membrane but fail to cross the inner membrane. Taking advantage of the high inner membrane potential gradient, lipophilic cations can easily accumulate within the mitochondria as well as permeate the phospholipid bilayers [29]. Vitamin E conjugated to TPP^+^ can accumulate into the mitochondria, where it decreases ROS more effectively than vitamin E alone [31], [52], and is able to ameliorate oxidative stress-mediated disease [15], [16].

While conjugating vitamin E to TPP^+^ has been previously described [53], our goal was to conjugate the vitamin E metabolite, α-CEHC, to TPP^+^ and to design a fast and efficient synthetic method using a lysine linker and solid phase synthesis. This method does not require isolation of synthetic intermediates, while reagents and by-products are washed away after each step. In addition, similar to trolox, α-CEHC contains the α-tocopherol ring structure but have a truncated side chain with one carbon longer than trolox [38]. The chroman ring of vitamin E becomes redox active at the mitochondria, where it forms semiquinone after detoxifying a free radical via hydrogen donation. The semiquinone is further reduced by intramitochondrial ascorbic acid or by electron donation [54]. The chroman ring is still intact in α-CEHC when conjugated to TPP^+^.

A lysine linker with two protecting groups (Fmoc and Mtt) was used, which enabled the conjugation of TPP^+^ and α-CEHC (Figure 1). The masked lysine was coupled onto the Rink Amide MBHA resin. HBTU and HOBt were used to enhance the coupling rate [55], [56], [57]. The Fmoc was then deprotected to allow for (3-carboxypropyl)TPP^+^ conjugation through its carboxylic acid group forming an amide bond. The Mtt protecting group was then removed. The removal of the protecting group enabled the carboxylic acid on α-CEHC side chain to form an amide bond with the lysine linker. The final product, TPP^+^-Lysine-α-CEHC (MitoCEHC), was then released from the resin via treatment with 95% TFA.

**Figure 2:**
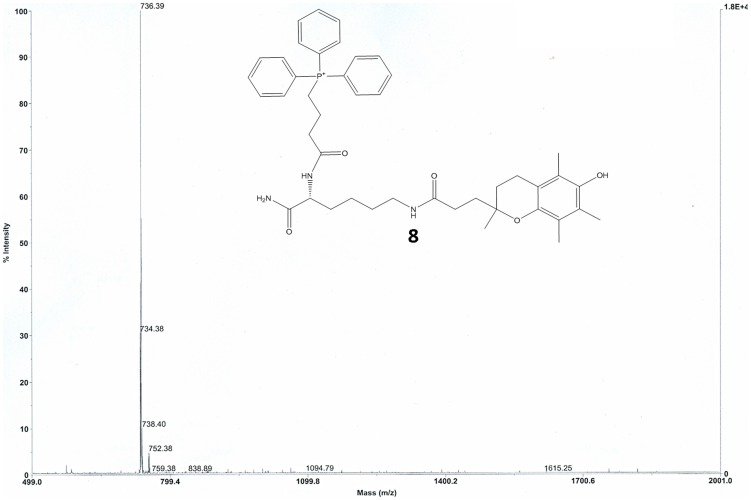
Mass Spectrometry and structure of MitoCEHC (8). The MALDI-TOF Mass Spectrometry of the final product from resin cleavage shows a molecular weight peak at 736.39 *m/z*. In addition, the structure of MitoCEHC (**8**) was created using ChemDraw Ultra software, with a calculated *m/z* for C_44_H_55_N_3_O_5_P^+^ of 736.39 (100%), which corresponds to the Mass Spectrometry results.

The final product was characterized by MALDI-TOF mass spectrometry (Figure 2). The molecular weight peak was at 736.39, which corresponds to the expected peak for the MitoCEHC generated by ChemDraw software (PerkinElmer Informatics, Cambridge, MA). The mass spectrometry data also shows virtually no trace of by-products, reagents or synthetic intermediates.

**Figure 3:**
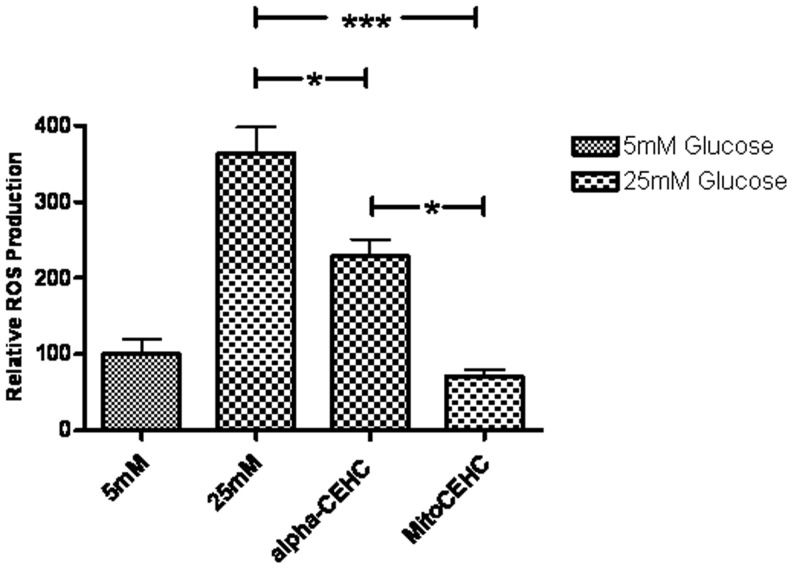
Effect of MitoCEHC on lowering ROS. ROS was measured via FACSCAN. The effect of 2 µM α-CEHC and 2 µM MitoCEHC on lowering ROS induced by high glucose in endothelial cells was tested 36 hours after treatment. MitoCEHC displays a higher significant effect on decreasing ROS than α-CEHC alone. Data are expressed as the percent of basal (5 mM glucose). Mean values were analyzed using one-way ANOVA with Tukey's posttest (*p<0.05, ***p<0.001).

The ability of final product (MitoCEHC) to diminish oxidative stress was examined *in vitro*. Oxidative stress is defined as the overproduction of oxidizing chemical species and the failure to eradicate their excess by enzymatic or non-enzymatic antioxidants. Elevation in ROS production is a factor in the etiology of cardiovascular disease by modifying lipids, proteins, and nucleic acids [58]. To further explore the antioxidant activity of the conjugated MitoCEHC, the oxidation (and hence fluorescence) of CM-H_2_DCFDA was measured (Figure 3). The H_2_DCFDA derivative with a thiol-reactive chloromethyl group was used due to its better retention in live cells than H_2_DCFDA. This derivative is retained better in cells because of its ability to bind covalently to intracellular components. BAEC were incubated with low (5 mM) and high (25 mM) glucose concentrations. The cells incubated under hyperglycemic conditions showed an increase in ROS production, which is mainly in the mitochondria [59]. Flow cytometry data also showed decrease in ROS production in the hyperglycemic cells treated with MitoCEHC. α-CEHC conjugated to TPP^+^ via a lysine linker (MitoCEHC) showed a stronger effect than α-CEHC alone (Figure 3). These results confirm the importance of mitochondria targeting as a strategy to diminish mitochondrial oxidative stress.

In an effort to investigate if the TPP^+^ conjugation to α-CEHC via a lysine linker would increase mitochondrial targeting, an *in vivo* experiment was performed. Since TPP^+^ conjugates are orally bioavailable when fed to mice [60], highly insulin resistant db/db mice were provided with 200 µM of the MitoCEHC in their drinking water. Although there is no direct correlation of dosing of vitamin E-like compounds between mice and humans, MitoCEHC doses selected in this study were based on maximal MitoVit E (vitamin E conjugated to TPP^+^) dosing of mice (500 µM) [45]. Therefore we chose the lowest dose (200 µM) that would still target the mitochondria for these studies. After two weeks of providing mice with MitoCEHC in their drinking water, their plasma was collected and hearts were harvested to isolate myocardial mitochondria. The isolated mitochondria were then lysed. The concentrations of MitoCEHC in the collected samples were simultaneously measured against a concentration standard curve. The retention times for the MitoCEHC standard and samples are shown as 13.9 and 13.6 minutes respectively (Figure S1). The MitoCEHC amount in the isolated mitochondria was 0.775±0.137 µg/0.1 g of mitochondria while the plasma concentration was 1.78±0.305 µg/ml. The untreated mice showed no trace of MitoCEHC in the isolated mitochondria or plasma. In addition to its antioxidant potential in cell lines, MitoCEHC accumulated in the mitochondria *in vivo*. More *in vivo* work still needs to be performed to test the effect of MitoCEHC on mitochondrial superoxide generation, oxygen consumption, and ATP production.

## Conclusion

In summary, the conjugation of α-CEHC to TPP^+^ was achieved using a fast and efficient method involving a lysine linker and solid phase synthesis. The conjugated product was effective in lowering oxidative stress in BAECs and targeting the mitochondria in type 2 diabetic db/db mice. The antioxidant effect of this drug may be clinically relevant and could be used to treat diseases related to oxidative stress such as cardiovascular disease. In this context, mitochondrial targeted versions of these antioxidants may provide a better protection against oxidative stress than untargeted ones. This chemistry also provides the framework for further products to be explored. TPP^+^ conjugation using this method should be investigated with other antioxidants such as co-enzyme Q and quercetin. Different amino acids could also be used as linkers to investigate the effect of their length (such as lysine versus glycine).

## Supporting Information

Figure S1 **LC/MS data showing the retention time of (A) sample from resin cleavage, MitoE (8) and (B) mitochondrial lysate of MitoE (8) treated mice.**
(TIF)
