# Supplementary figures and images for: Solid Phase Synthesis of Mitochondrial Triphenylphosphonium-Vitamin E Metabolite Using a Lysine Linker for Reversal of Oxidative Stress
Source: PLoS One. 2013 Jan 14;8(1):e53272. doi: 10.1371/journal.pone.0053272 (PMC3544826; doi:10.1371/journal.pone.0053272)

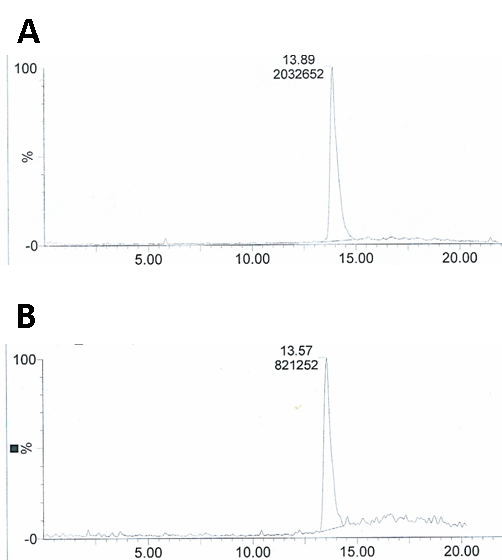

Supplement: Figure S1 — LC/MS data showing the retention time of (A) sample from resin cleavage, MitoE (8) and (B) mitochondrial lysate of MitoE (8) treated mice. (TIF) [file pone.0053272.s001.tif]
